# Lay support for pregnant women with social risk: a randomised controlled trial

**DOI:** 10.1136/bmjopen-2015-009203

**Published:** 2016-03-02

**Authors:** Sara Kenyon, Kate Jolly, Karla Hemming, Lucy Hope, Jackie Blissett, Sophie-Anna Dann, Richard Lilford, Christine MacArthur

**Affiliations:** 1 Institute of Applied Health Research, College of Medical and Dental Sciences University of Birmingham, Birmingham, UK; 2 University of Worcester; 3 School of Psychology, University of Birmingham, Birmingham, UK; 4 Department of Population Evidence & Technologies, University of Warwick, Warwick Medical School, Coventry, UK

**Keywords:** PUBLIC HEALTH, OBSTETRICS, pregnancy, social/lay support

## Abstract

**Objectives:**

We sought evidence of effectiveness of lay support to improve maternal and child outcomes in disadvantaged families.

**Design:**

Prospective, pragmatic, individually randomised controlled trial.

**Setting:**

3 Maternity Trusts in West Midlands, UK.

**Participants:**

Following routine midwife systematic assessment of social risk factors, 1324 nulliparous women were assigned, using telephone randomisation, to standard maternity care, or addition of referral to a Pregnancy Outreach Worker (POW) service. Those under 16 years and teenagers recruited to the Family Nurse Partnership trial were excluded.

**Interventions:**

POWs were trained to provide individual support and case management for the women including home visiting from randomisation to 6 weeks after birth. Standard maternity care (control) included provision for referring women with social risk factors to specialist midwifery services, available to both arms.

**Main outcome measures:**

Primary outcomes were antenatal visits attended and Edinburgh Postnatal Depression Scale (EPDS) 8–12 weeks postpartum. Prespecified, powered, subgroup comparison was among women with 2 or more social risks. Secondary outcomes included maternal and neonatal birth outcomes; maternal self-efficacy, and mother-to-infant bonding at 8–12 weeks; child development assessment at 6 weeks, breastfeeding at 6 weeks, and immunisation uptake at 4 months, all collected from routine child health systems.

**Results:**

Antenatal attendances were high in the standard care control and did not increase further with addition of the POW intervention (10.1 vs 10.1 (mean difference; MD) −0.00, 95% CI (95% CI −0.37 to 0.37)). In the powered subgroup of women with 2 or more social risk factors, mean EPDS (MD −0.79 (95% CI −1.56 to −0.02) was significantly better, although for all women recruited, no significant differences were seen (MD −0.59 (95% CI −1.24 to 0.06). Mother-to-infant bonding was significantly better in the intervention group for all women (MD −0.30 (95% CI −0.61 to −0.00) p=0.05), and there were no differences in other secondary outcomes.

**Conclusions:**

This trial demonstrates differences in depressive symptomatology with addition of the POW service in the powered subgroup of women with 2 or more social risk factors. Addition to existing evidence indicates benefit from lay interventions in preventing postnatal depression. This finding is important for women and their families given the known effect of maternal depression on longer term childhood outcomes.

**Trial registration number:**

ISRCTN35027323; Results.

**Strengths and limitations of this study**

- Large, robust, individual, randomised controlled trial to evaluate a real National Health Service service demonstrating differences in aspects of maternal psychological health from the addition of lay workers.
- Achieved excellent follow-up of both primary outcomes in intervention and control arms which is unusual in studies of women with social disadvantage.
- Baseline Edinburgh Postnatal Depression Scale scores not feasible in real service pragmatic trial, so change in score not obtained, but routine baseline data on current or previous mental health problem was the same across trial arms.
- Set within the UK maternity care system where standard care includes specialist midwife referral for women with some social risks, so effect of intervention may be greater in maternity systems without this.

## Background

Postnatal depression is a major public health issue, with lasting effects on the child,^1–3^ and meta-analyses have reported prevalences of 13% and 19%.^4–6^ Women with antenatal depression, or with a previous history, are at higher risk,^7^ but most pregnant women who go on to have postnatal depression do not have these risk factors. It is known that postnatal depression is associated with social isolation and inadequate support.^8^ Many of the factors considered to be indicators of increased risk of adverse perinatal morbidity and mortality are also surrogates for social isolation, including teenage pregnancy,^9^ minority ethnic group,^10^ ^11^ experience of domestic violence,^12^ asylum seekers and refugees,^13^ and homelessness.^14^

A Cochrane review^15^ showed that taken as a group, psychosocial and psychological interventions were more effective in preventing postnatal depression than usual care, but there is little evidence regarding lay support except among women screened positive for possible depression. A recent synthesis of barriers to engagement with maternity services in women with social disadvantage^16^ suggested that lay workers providing non-judgemental support, working in conjunction with antenatal services, would be well received by women, however, evidence on effectiveness is lacking. The need to provide additional lay support (in this instance Pregnancy Outreach Workers, POWs) to women with identified social risk factors had been recognised in the West Midlands, and a service developed. The hypothesis was that this might improve engagement with maternity services (and thereby improve maternal and neonatal birth outcomes), and reduce postnatal depression, and we undertook a pragmatic randomised controlled trial to evaluate this.

## Methods

### Design

The study was a pragmatic, individually randomised, controlled trial across a UK geographical area containing three maternity units, where social risk factors are systematically identified at routine midwife antenatal booking. Nulliparous women under 28 weeks gestation, with social risk factors, were eligible. Exclusions were women under 16 years of age and teenagers already recruited to the Family Nurse Partnership Trial. Multiparous women were not included since some social support was already available for this group which could have masked a trial effect. Potentially eligible women (ie, nulliparous women with one or more social risk factor) were identified at midwife antenatal booking and given information about the trial. Following agreement, they were referred to specifically trained midwives who obtained informed consent, and randomised women. Randomisation to standard maternity care, or addition of the POW service was by telephone using a registered trials unit. Randomisation lists were computer generated (by trial statistician) using random block sizes (4–12) and stratified for Maternity Trust.

POWs were trained to provide individual case management for the women including home visits, and were integrated into the community midwifery teams. Objectives were to encourage women to attend antenatal appointments, make healthy lifestyle choices, to provide social/emotional support, and help ensure benefits, housing difficulties and mental health problems were managed. In the postnatal period (to 6 weeks postpartum), POWs also provided breast feeding and advice about infant care. The POW service was developed before the trial began, but not available outside the trial, and was provided by an independent organisation, who had access to supervision from experts with specific skills and knowledge.

Standard UK maternity care (control) included provision for referring women with social risk factors to specialist midwives or directing them to other agencies but did not include the offer of the POW service.

### Outcome measures, data collection and follow-up

The two primary outcomes were engagement with antenatal care and maternal postnatal depression 8–12 weeks after birth. Antenatal attendance was assessed by number of antenatal visits attended, including all visits with a healthcare professional (midwife, obstetrician, mental health specialist) in hospital or community except for routine dating and abnormality scans. Maternal depression was assessed using the Edinburgh Postnatal Depression Scale^17^ (EPDS) at 8–12 weeks postpartum by postal/telephone questionnaire. We chose EPDS as it is the most commonly used validated instrument to assess postpartum symptoms. The original Cox publication^17^ quotes a cut-off score as ≥13, so we present data for that.

### Secondary outcomes

*Maternal and neonatal birth outcomes* included routinely collected birth outcome data detailed in online supplementary material. Data to evaluate other maternal psychological outcomes (self-efficacy and bonding) were collected using validated tools 8–12 weeks postpartum (Pearlin and Schooler Mastery Scale^18^ and Mother-to-infant Bonding Scale^19^).

*Longer term infant outcomes*: attendance at child development assessments and breastfeeding at 6 weeks and immunisation uptake at 4 months were collected from routine child health systems (detailed in see online supplementary information).

10.1136/bmjopen-2015-009203.supp1 Supplementary data

### Data collection

Collection of demographic data, gestation, ethnicity, medical history at booking and systematically assessed social risk factors (table 1) were part of midwife routine antenatal booking information and available for trial use. Blinding of women and caregivers was not possible, but those who collected/entered data remained blind to allocation.

**Table 1:** Baseline characteristics and description of social risk factors identified at randomisation

Baseline characteristics	POW n=662	Standard care n=662
*Maternal age (years) median, IQR*	21.8 (19.0, 25.5)	21.5 (18.8, 24.6)
*Gestation at recruitment*		
Median, IQR	12.9 (9.9, 17.3)	12.7 (9.9, 18.0)
Under 12 weeks	288 (43)	283 (43)
12–19+6 weeks	266 (40)	252 (38)
≥20 weeks	108 (16)	127 (19)
*Ethnicity*		
Africa (North Africa, sub-Sahara, other)	41 (6)	46 (8)
Asia		
Pakistan	100 (15)	107 (16)
India	26 (4)	27 (4)
Bangladesh	24 (4)	19 (3)
Other	22 (3)	16 (2)
Caribbean	24 (4)	45 (7)
European		
Britain	320 (48)	315 (48)
Eastern Europe	23 (4)	20 (3)
Other	6 (1)	6 (1)
Middle East	23 (4)	19 (3)
Other	53 (8)	42 (6)
*Index of multiple deprivation from postcode at recruitment*		
Quintile 1	494 (75)	488 (74)
Quintile 2	99 (15)	110 (17)
Quintile 3	51 (8)	49 (7)
Quintile 4	15 (2)	13 (2)
Quintile 5	3 (<1)	2 (<1)
*Medical history noted at booking*	320 (48)	301 (45)
*Social risk factor*		
Housing problems such as rent arrears, temporary accommodation registered with National Asylum Support Service (NASS) or of No Fixed Abode (NFA)	282 (43)	262 (40)
Teen parent (under 20 years old)	230 (35)	249 (38)
Smoking	192 (29)	183 (28)
Difficulty with the English language both spoken and written	176 (27)	169 (26)
Identified benefit problem	154 (23)	160 (24)
UK resident for under a year	116 (18)	93 (14)
Clinical diagnosis of past or present mental illness	100 (15)	96 (15)
No support from either partner or family or friend	63 (10)	80 (12)
Body mass index ≥35	34 (5)	33 (5)
Body mass index ≤18	32 (5)	26 (4)
Late booking (defined as booking after 18 weeks gestation)	28 (4)	31 (5)
Woman/household member in receipt of social services support, including child protection	24 (4)	34 (5)
Drug misuse including other's in the household	19 (3)	17 (3)
Domestic abuse	13 (2)	19 (3)
Alcohol misuse	6 (1)	7 (1)
DNA 2 or more antenatal appointments (under 28 weeks gestation)	5 (1)	8 (1)
*Number of social risk factors identified*		
1 social risk factor	174 (26)	194 (29)
2 social risk factors	269 (41)	247 (37)
3 social risk factors	141 (21)	145 (22)
4 or more social risk factors	78 (12)	76 (11)

Values are numbers (percentages) unless otherwise stated.

POW, Pregnancy Outreach Worker.

*Maternal and neonatal birth outcome data* were obtained from hospital systems. Number of antenatal contacts was not recorded electronically, so was collected by hand-abstraction from notes.

*Maternal psychological outcomes* were obtained from a postal questionnaire sent at 8–12 weeks postpartum using methods shown to maximise response rates.^20^ Women could opt to complete the questionnaire by phone, and interpreters were available. Details of these and the data quality checks are given in online supplementary material. Data on POW contacts, collected by the independent service and checked by the researchers, are shown in the online supplementary material.

### Study oversight

The trial was not registered with the controlled trials register until after first patient recruitment. The trial was a pragmatic one to evaluate a real-time National Health Service implementation, so evaluation had to take place urgently, otherwise trial design would have been compromised. We were informed at that time that only CTIMP trials required trial registration prior to first patient enrolment. Our trial documentation is available for scrutiny, which provides evidence that this did not compromise our research probity.

### Sample size justification

The sample size was 421 women per arm to provide 90% power (5% significance level) to detect 1.5 mean EPDS score reduction (SD 6), and provide greater than 90% power to detect 1.5–2 increased antenatal contacts (SD 6) allowing for 20% drop-out or loss to follow-up (detailed sample size rationale in published protocol).^21^

Prior to the trial, data was not available on numbers of social risk factors women had. Following a successful initial 6 months pilot where 475 women were recruited, it was observed that 64% had two or more social risk factors, and it was agreed to increase the sample size to allow power to detect differences in primary outcomes in the prespecified subgroup of women with two or more social risk factors, that is, so that the required sample size of 421 would be recruited within this subgroup. This powered subgroup gave a sample size of 658 women per arm.

### Statistical analysis

Baseline characteristics were summarised by control and intervention arms using means and SDs, medians and inter-quartile ranges, or numbers and percentages, as appropriate. For continuous outcomes, we reported mean (SE) in each arm, and mean difference. For continuous variables, statistical significance was assessed using two sample t tests assuming equal variances, or a Mann-Whitney U test, as appropriate. For binary outcomes, we reported the number (percentage) in each arm, along with relative risk (RR) and number needed to treat, calculated as 1 divided by risk difference; and also the risk difference (RD). For RRs, we calculated 95% CIs using standard normal approximation methods, and tested for statistical significance using χ^2^ test. Analyses were carried out in Stata V.12, according to intention to treat principles, and included women for whom outcomes could be collected. Analyses of primary outcomes were replicated independently. We undertook additional analysis of EPDS score ≥13 as a binary outcome^17^ to enable comparisons with other trials.

## Results

### Women and follow-up

Between July 2010 and October 2011, 1324 nulliparous women with identified social risk factors were randomised, 662 to standard maternity care and 662 to addition of the POW service. Follow-up data collection, which included both postal questionnaire and longer term infant outcomes data were completed by September 2013.

**Figure 1:**
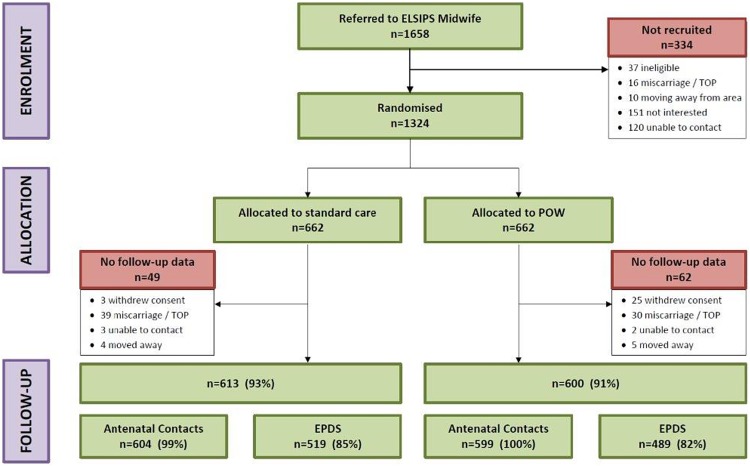
Consort diagram. EPDS, Edinburgh Postnatal Depression Scale.

Baseline characteristics were similar between groups including identified social risk factors (Table 1). Of women allocated standard care, 49 were not included in analyses (39 had subsequent miscarriage/termination, so no outcomes), and in those allocated POW service, 62 women were not included (30 had subsequent miscarriage/termination) (figure 1). Analyses, therefore, included 613 women allocated standard care, and 600 allocated the POW service. Primary outcome data regarding antenatal contacts were available for 99% of women in the standard care, and 100% in the POW service arms. Data from the questionnaire on EPDS 8–12 weeks postpartum was available for 85% and 82% of groups, respectively: 180 women completed the questionnaire via an interpreter, and 146 in English by phone as requested by the women.

Baseline characteristics of responders and non-responders to the postnatal questionnaire were broadly similar although marginally more non-responders were younger, recruited at earlier gestation, had housing problems, or were smokers (table 2).

**Table 2:** Baseline characteristics for responders and non-responders to the questionnaire

	Responders n=1008 (83%)	Non-responders n=205 (17%)
*Maternal age (years) median, IQR*	21.0 (21.98, 22.58)	19.0 (20.48, 21.70)
*Gestation at recruitment*		
Median, IQR	13.2 (14.05, 14.78)	12.6 (13.18, 14.59)
Under 12 weeks	421 (41)	92 (45)
12–19+6 weeks	387 (38)	84 (41)
≥20 weeks	200 (20)	29 (14)
*Ethnicity*		
Africa (North Africa, sub-Sahara, other)	68 (7)	12 (6)
Asia		
Pakistan	163 (16)	26 (13)
India	41 (4)	4 (2)
Bangladesh	34 (3)	5 (2)
Other	34 (3)	2 (1)
Caribbean	53 (5)	12 (6)
European		
Britain	475 (47)	110 (54)
Eastern Europe	35 (3)	5 (2)
Other	9 (0.9)	1 (1)
Middle East	30 (3)	6 (3)
Other	66 (7)	21 (10)
*Index of multiple deprivation from postcode at recruitment*		
Quintile 1	750 (74)	143 (70)
Quintile 2	151 (15)	40 (20)
Quintile 3	81 (8)	17 (8)
Quintile 4	22 (2)	5 (2)
Quintile 5	4 (<0.5)	NA
*Medical history noted at booking*	687 (68)	140 (68)
*Social risk factors*		
Housing problems such as rent arrears, temporary accommodation registered with National Asylum Support Service (NASS) or of No Fixed Abode (NFA)	392 (39)	107 (52)
Teen parent (under 20 years old)	339 (34)	105 (51)
Smoking	269 (27)	75 (37)
Difficulty with the English language both spoken and written	277 (28)	40 (20)
Identified benefit problem	244 (24)	51 (25)
UK resident for under a year	166 (17)	24 (12)
Clinical diagnosis of past or present mental illness	152 (15)	30 (15)
No support from either partner or family or friend	99 (10)	28 (14)
Body mass index ≥35	514 (5)	11 (5)
Body mass index ≤18	46 (5)	10 (5)
Late booking (defined as booking after 18 weeks gestation)	52 (5)	7 (3)
Woman/household member in receipt of social services support, including child protection	37 (4)	16 (8)
Drug misuse including other's in the household	23 (2)	9 (4)
Domestic abuse	27 (3)	5 (2)
Alcohol misuse	11 (1)	1 (0.5)
DNA 2 or more antenatal appointments (under 28 weeks gestation)	7 (0.7)	5 (2)
*Social risk identified*		
0 social risk factor	1 (<0.5)	NA
1 social risk factor	258 (26)	55 (27)
2 social risk factors	384 (38)	70 (34)
3 social risk factors	229 (23)	50 (24)
4 or more social risk factors	136 (14)	30 (15)

Values are numbers (percentages) unless otherwise stated.

NA, not applicable.

### Primary outcomes and prespecified subgroup comparisons

Antenatal attendance: No difference was seen between groups, either for all women or for women with two or more social risk factors (table 3).

**Table 3:** Primary outcomes and prespecified subgroup analysis

Antenatal attendance	POW n=599	Standard care n=604	Mean difference (95% CI)	p Value	
Number of contacts, mean (SE)	10.1 (0.14)	10.1 (0.13)	−0.00 (−0.37 to 0.37)	0.99	
Number with ≥10 contacts	322 (54.3)	320 (53.5)	RR=1.01 (0.91 to 1.13)	0.78	
*Number of social risk factors*					
1 social risk factor	9.9 (0.27) n=152	10.0 (0.23) n=173	−0.19 (−0.89 to 0.51)	0.59	
2 or more social risk factors	10.2 (0.16) n=440	10.1 (0.15) n=425	0.06 (−0.37 to 0.50)	0.82	
**EPDS**	**POW** **n=489 (49)**	**Standard Care n=519 (51)**	**Mean Difference (95% CI)**	**p Value**	**NNT**
Mean, SE	6.76 (0.23)	7.35 (0.24)	−0.59 (−1.24 to 0.06)	0.08	
EPDS≥13	61 (12)	87 (17)	RR=0.74 (0.55 to 1.01)	0.05	23
*Number of social risk factors*					
1 social risk factor	n=128	n=159			
Mean, SE	6.8 (0.48)	6.9 (0.42)	−0.14 (−1.38 to 1.10)	0.82	
EPDS≥13	13 (10)	24 (15)	RR=0.67 (0.36 to 1.27)	0.21	20
2 or more social risk factors	n=361	n=360			
Mean, SE	6.8 (0.27)	7.6 (0.29)	−0.79 (−1.56 to −0.02)	0.05	
EPDS≥13	48 (13)	63 (18)	RR=0.76 (0.54, 1.07)	0.12	24

Values are numbers (%) unless otherwise stated.

EPDS, Edinburgh Postnatal Depression Scale; NNT, number to treat; POW, pregnancy outreach worker.

Postnatal depression: The prespecified comparison for women with two or more social risk factors showed a statistically significant reduction in mean EPDS (MD −0.79 (95% CI −1.56 to −0.02) p=0.05), although no significant differences were seen in the mean EPDS (mean difference (MD) −0.59 (95% CI −1.24 to 0.06)) for all the women recruited. The additional analysis of EPDS as a binary outcome showed a relative risk reduction of 26% for those with an EPDS ≥13 (RR 0.74 (95% CI 0.55 to 1.01) p=0.05), which equates to a reduction of five percentage points (17% vs 12%, RD 0.04 (95% CI −0.00 to 0.09)). In the group with two or more social risk factors, there was a reduction of five percentage points for EPDS ≥13 (18% vs 13%), giving an RD −0.04 (95% CI −0.09 to 0.01) and RR 0.76 (95% CI 0.54 to 1.07).

### Secondary outcomes

#### Maternal and infant outcome data

No differences were seen in any secondary maternal or neonatal birth outcomes, including the adverse perinatal composite outcome (see online supplementary tables S1 and S2).

10.1136/bmjopen-2015-009203.supp2 Supplementary tables

Mother-to-infant bonding was significantly better in the intervention group for all women (MD −0.30 (95% CI −0.61 to 0.00) p=0.05), but did not achieve statistical significance for those with two or more social risk factors (MD −0.35 (95% CI −0.72 to 0.01) p=0.06). Maternal self-efficacy was higher, but not significantly so, in both the intervention group overall (MD 0.43 (95% CI −0.06 to 0.91) p=0.08) and in the group with two or more social risks (MD 0.48 (95% CI −0.08 to 1.04) p=0.09) (see online supplementary table S3). Routine child assessment attendance, primary immunisation uptake, and breastfeeding at 6–8 weeks did not differ between groups (see online supplementary table S4).

#### Description of POW service

Data on intensity of the POW service showed over 17 000 contacts between POWs and women, 27% of which were face to face, with half of them lasting 1–2 h (see online supplementary table S5). Most contacts took place antenatally (77%). The most common type of support recorded as given by the POWs (see online supplementary table S6) were finance/legal/benefits (19%), emotional and health matters (17%) and housing (15%). Additional social risk factors were disclosed to the POWs after recruitment by 83 women, most commonly; social service/child protection 35; domestic abuse 30; housing problems 21.

## Discussion

Despite prior indication of local low engagement with maternity care services in disadvantaged women, no difference in antenatal contacts was identified between trial groups. This was at the UK recommended level of 10^22^ visits in both groups. Various other initiatives to encourage antenatal attendance and engagement had already occurred, thereby reducing potential for further improvement. Since antenatal attendance was unaffected, it is not surprising that maternal and neonatal birth outcomes were no different between trial groups.

This trial, however, provides some evidence of a benefit of lay support to maternal depression in women with social risk factors relative to similar controls: while there was no significant difference in mean EPDS for the intervention group as a whole, there was a significant difference in the powered subgroup of women with two or more social risk factors, and mother-to-infant bonding scores were better than among controls overall. Systematic reviews show that children of depressed mothers are more likely to suffer insecure attachment, behavioural problems, cognitive developmental deficits and difficulties in emotional functioning, with impaired bonding between mother and child.^23^ The implications of a reduction in maternal depression are likely to be of lasting importance to the child, family and more generally to society.

The strengths of this trial are that it was an evaluation of an existing service using highest quality methodology with excellent balance between groups, including social risks, and it achieved excellent retention and follow-up which is uncommon among disadvantaged women. A possible limitation is that EPDS was not administered at baseline, but this was not feasible as a pragmatic trial evaluating a real service with inclusion based on routine maternity booking information. The difference in maternal depression we have seen might have been influenced by baseline differences in previous or current mental health problems, but prevalence of this was the same at 15% in the trial groups. Our results could also have been influenced by the fact that 25 women recruited to the intervention group subsequently withdrew relative to only two in the control group, however, this was almost entirely a result of the women deciding that they did not want to continue with the POW service after meeting their POW, and not unsurprising within a real service situation.

Improvements in aspects of maternal psychological health in women who received support from the POWs are plausible. For any service-level intervention to be effective it must be implemented and must show impact on the short-term factors that mediate improved long-term outcomes on the service user.^24^ In the case of the POW service, we have evidence that the service was implemented with commitment: there was an average of over six face-to-face contacts per woman, over half of which exceeded 1 h, and an overall average of more than 24 total contacts per woman. The ingredients shown in the literature to characterise an effective service, practical and emotional support,^25^ were also provided, and evidence that the POWs achieved positive relationships with women comes from the observation that many divulged sensitive information, for example, domestic abuse.

**Figure 2:**
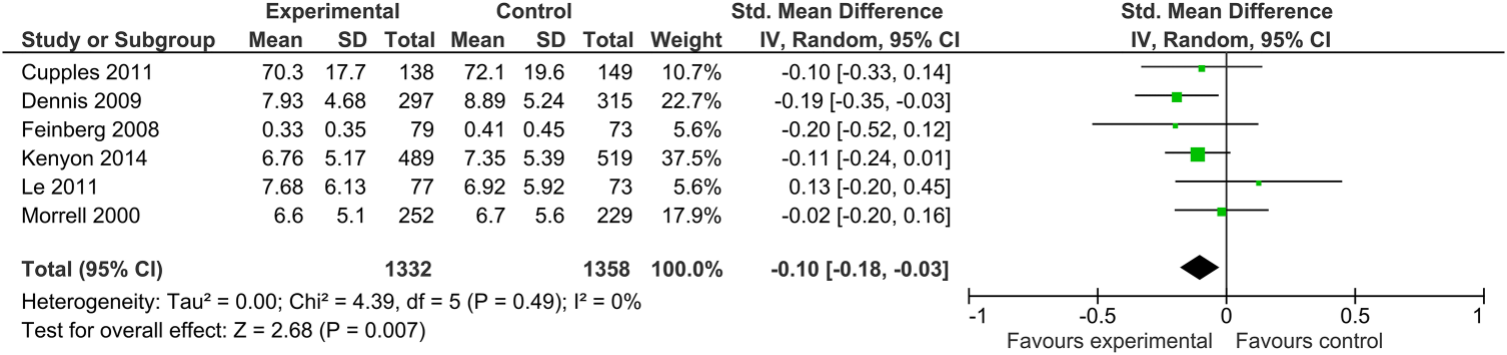
Forest plot of difference in mean depression scores at final study assessment in lay-based interventions. Two of the seven lay worker trials in the Cochrane review did not report mean depression score at final study assessment. One small trial (n=65) of women screened positive for probable depression reported depression diagnosis and showed a reduction with lay support, and the other in India (n=468) reported depressive symptomatology and showed no difference.

The Cochrane review of ‘Psychosocial and psychological interventions for preventing postpartum depression’^15^ identified 28 trials, involving almost 17 000 women with types of intervention divided into psychological (eg, debriefing, cognitive behavioural therapy) and psychosocial interventions (eg, antenatal/postnatal groups, professional/lay home visits). The review concluded that as a group these interventions significantly reduced the development of postpartum depression. However, only seven trials were of lay interventions, three of which recruited women screened positive for probable depression, and none of the remaining four trials were effective in preventing postnatal depression. No difference in mean depression scores at final study assessment overall was seen in the lay support trials (MD −10 (−0.20 to 0.01)), and the review recommended further trials of support by lay individuals. Addition of data from our trial to this meta-analysis indicates a significant reduction in mean depression scores MD −0.10 (−0.18 to −0.03) in lay support trials (figure 2). Before our trial, therefore, evidence was inconclusive on whether postnatal depression could be prevented through a lay-based intervention, except among women already exhibiting possible depression.

Given that UK standard maternity care routinely provides some specialist services to support women with social risks, in international contexts where such standard services are lacking, benefit from a similar POW service might be greater than evidenced here. Moreover, this trial only included nulliparous women, and it is plausible that the effect of the service may be greater in multiparous women, likely to have more social risks. This trial provides evidence that a lay support service targeted to women with two or more social risk factors improves aspects of maternal psychological health relative to controls; such improvements are likely to be of lasting impact due to the known effect of maternal depression and poor attachment on longer term childhood outcomes. This, together with the relatively low costs of the service (approximately £500 000 for 1000 women annually), means that consideration should be given by policymakers to introduction of a lay support service.
