# Supplementary material for: Lay support for pregnant women with social risk: a randomised controlled trial
Source: BMJ Open. 2016 Mar 2;6(3):e009203. doi: 10.1136/bmjopen-2015-009203 (PMC4785315; doi:10.1136/bmjopen-2015-009203)
Supplement: Supplementary data [file bmjopen-2015-009203supp.pdf]

Supplementary information - Lay support for pregnant women with social risk: randomised controlled trial

Maternal outcomes included: length of labour (first, second and third stages); mode of birth (spontaneous vaginal birth, instrumental birth or caesarean section); perineal trauma (episiotomy, degree of laceration); incidence of maternal morbidity (e.g. postpartum haemorrhage, shoulder dystocia); length of stay in hospital.

Neonatal outcomes included: composite outcome of adverse perinatal outcome (comprising perinatal mortality, preterm birth before 34 weeks, birth weight 10th centile or below and admission to neonatal unit); Apgar score at 5 minutes; arterial cord blood gases, if taken; birth weight, breastfeeding initiation rate; length of stay in hospital; and for those admitted to the Neonatal Unit, if applicable: oxygen at 36 weeks post conceptual age, retinopathy of prematurity, abnormal cerebral ultrasound prior to discharge (e.g., intraparenchymal cerebral bleed, hydrocephalus, parenchymal cysts), necrotising enterocolitis (Bells Stage I, II or III), and culture positive sepsis requiring greater than 5 days antibiotic treatment.

Primary immunisation of babies in the UK at the time of the study involved a single injection at 2, 3 and 4 months. This included diphtheria, tetanus, pertussis, polio, *Haemophilus influenzae* type b (Hib) on all occasions, with the addition of pneumococcal infection at two months, meningitis C at three months and both at four months. We report complete immunisation if all were recorded as given, partial if any component was not given and none if there was no record of primary immunisation.

Details of methods employed to maximise postal questionnaire response rates

Women were sent a card to congratulate them on the birth of their baby, and reminding them of the study in which they had agreed to participate. The family doctor was contacted to ensure that on–going contact was appropriate. Women were then either sent a questionnaire (containing a voucher for £5) or contacted by telephone, according to the preference expressed when they agreed to

participate in the study. If no response was obtained a reminder phone call, text or letter was sent, as applicable. Contact details were checked with the local research midwife and final contact made by the research midwife or lead healthcare professional, as appropriate. Women whose babies were on Neonatal Intensive Care Unit (NICU) or had died were included (appropriate letters were sent), but no reminders were sent to the women whose babes had died.

Those involved in data collection and entry remained blind to allocation.

Process outcomes were collected systematically by the POWs and entered on a bespoke database.

Data quality checks undertaken

Birth outcome data was transferred electronically from the provider units to the registered trials unit once the women had given birth. An audit was undertaken of a random 20% of women to compare the data that had been transferred electronically with the maternity notes and data items found to be missing or inaccurate in >5% of cases were collected directly from maternity notes. These included; length of labour (36% inaccurate), perineal trauma (13% inaccurate) including episiotomy (5% inaccurate), mode of birth (8% inaccurate), estimated blood loss (34% inaccurate), length of stay in hospital (13% inaccurate), and whether the baby had been admitted to an NICU (8% inaccurate). This data therefore had to be collected from the maternity notes for the whole sample.

Data for the primary outcomes (antenatal attendance and EPDS) were double entered. EPDS was also re–calculated for 20% of women to ensure it had been scored correctly (100% correct). The accuracy of the entry of birth data was checked in a random sample of 20% of women and minimal errors found (99.8% correct).

Baseline and questionnaire data was checked in a 20% sample. Minimal errors were found (99.6% correct) and over half of the errors were found in the questionnaire data. Therefore data from the

questionnaires on self–efficacy and mother–to–infant bonding was visually double–checked for all women.

The completeness of data regarding use of the POW service was checked for 80% of women (number and type of POW contacts) against Gateway's records for all women to ensure complete record of contacts was held. All instances of additional social risk disclosure were double–checked. Lists were supplied to Gateway to manually check any of the following: no support given for previously disclosed social risk, lack of breast/bottle feeding support, lack of baby care. The accuracy of data entry of the POW data was visually double–checked in a random sample of 20% of women, and minimal errors found.

The *Theme Management Group* (TMG) monitored progress and consisted of *University of Birmingham;* Richard Lilford (Director of CLAHRC BBC Programme), Jackie Blissett (Reader in Psychology), Nicola Gale (Senior Research Fellow in Medical Sociology), Caroline Fox (Lecturer in Obstetrics and Gynaecology); *Gateway Family Services;* Vicki Fitzgerald (Chief Executive, Gateway Family Services), Joanne Harper (Programme Lead, Gateway Family Services); *Primary Care Trusts;* Jacky Chambers (Director of Public Health, HoBtPCT), Diane Reeves (Lead for Birmingham Health and Wellbeing Partnership) or their nominated representatives; Heads of Midwifery; Jenny Henry (Birmingham Women's NHS Foundation Trust), Elaine Newell (Sandwell & West Birmingham NHS Trust) and Paula East (Heart of England NHS Foundation Trust); Consumers; Amy Mclean and Michelle Dalton, Maternity Services Liaison Committee and Ishrat Nasim and Zainab Sesay, (POW users).

Independent oversight to the trial was provided by the CLAHRC Steering Group which comprised of Rashmi Shukla (Chair), Regional Director of Public Health/Medical Director NHS West Midlands & Department of Health Government Office for the West Midlands; Diane Reeves, Medical Director (Commissioning), South Birmingham PCT; David Adams, University of Birmingham; Robert Bacon,

Chief Executive, Sandwell PCT; Barry Clark, Chaplaincy Manager, UHBFT; Jo Foster, Academy Manager, BCRA; Richard Lilford, Director CLAHRC & Director BCRA; Julian Miller, Head of Financial Management and Planning, UHBFT; David Rosser, Medical Director, UHBFT; Michael Sheppard, Vice-Principal, University of Birmingham.
