# Supplementary material for: Lay support for pregnant women with social risk: a randomised controlled trial
Source: BMJ Open. 2016 Mar 2;6(3):e009203. doi: 10.1136/bmjopen-2015-009203 (PMC4785315; doi:10.1136/bmjopen-2015-009203)
Supplement: Supplementary tables [file bmjopen-2015-009203supp_tables.pdf]

Supplementary tables – Lay support for pregnant women with social risk: randomised controlled trial

Supplementary Table 1: Maternal birth secondary outcomes

	POW n=600, 606 infants (6 multiples)	Standard Care n=613, 619 infants (6 multiples)	Mean Difference (95% CI)	P=
Length of labour (onset to time of birth) mins[&]	n=595	n=603		
Median (IQR)	269 [115, 531]	272 [95, 501]	NA	0·39
Mode of birth[&]	n=604	n=616	NA	0·72
Spontaneous vaginal birth	353 (58%)	347 (56%)		
Instrumental birth				
Forceps	55 (9%)	58 (9%)		
Ventouse	57 (9%)	50 (8%)		
Caesarean section				
Elective	17 (3%)	19 (3%)		
Emergency	122 (20%)	142 (23%)		
Estimated blood loss	n=596	n=609	NA	0·31
Up to 500mls	459 (77%)	446 (73%)		
500–1000mls	119 (20%)	140 (23%)		
>1000mls	18 (3%)	23 (4%)		
Perineal trauma	342 (57%)	334 (54%)	2.4 (–3.2, 8.1)	0·39
Degree of laceration	n=220*	n=210	NA	0·56
First degree	42 (19%)	48 (23%)		
Second degree	161 (73%)	147 (70%)		
Third or fourth degree	16 (8%)	15 (7%)		
Incidence of maternal morbidity				
Episiotomy	152/598 (25%)	144/609 (24%)	1·8 (–3·0, 6.7)	0·45
Postpartum haemorrhage	137/596 (23%)	162/610 (27%)	–3·6 (–8·4, 1·3)	0·15
Maternal admission to HDU/ITU	21/597 (3%)	24/608 (4%)	–0·4 (–2·6, 1·7)	0·70
Hysterectomy	1/598 (<1%)	0/610 (0%)	0·2 (–0.2, 0.1)	0·31
Uterine Rupture	0/598 (0%)	0/610 (0%)	NA	NA
Shoulder dystocia	4/598 (1%)	3/610 (<1%)	–0·2 (–0·7, 0·1	0·68
Length of stay in hospital (days)				
Median (IQR)	2 [1,4]	2 [1,4]	NA	0·84

*One un–specified degree; NA either no events or non-parametric test (Rank sum). Values are numbers (percentages) unless otherwise stated.

Supplementary Table 2: Neonatal outcomes

**Pre–specified perinatal composite outcome**	**POW n=604**	**Standard Care n=616**	**Relative risk (95% CI)**	**P=**
Perinatal mortality	6 (1%)	3 (<1%)	2·04 (0·51, 8·12)	0·30
Preterm birth before 34 weeks	20 (3%)	19 (3%)	1·07 (0·58, 1·99)	0·82
Birth weight 10th centile or below	127 (21%)	141 (23%)	0·92 (0·74, 1·14)	0·43
Admission to neonatal unit	77 (13%)	81 (13%)	0·97 (0·72, 1·30)	0·83
Any outcome	180 (30%)	204 (30%)	0·90 (0·76, 1·06)	0·24
**2 or more social risk factors**				
Perinatal mortality	4 (1%)	2 (<1%)	1·97(0·36, 10·71)	0·42
Preterm birth before 34 weeks	14 (3%)	16 (4%)	0·86 (0·43, 1·75)	0·68
Birth weight 10th centile or below	95 (21%)	106 (24%)	0·88 (0·69, 1·13)	0·32
Admission to neonatal unit	58 (13%)	62 (14%)	0·92 (0·66, 1·29)	0·64
Any outcome	133 (30%)	153 (35%)	0·86 (0·71, 1·03)	0·11

NB: Values are numbers (%)

**Neonatal secondary outcomes**	**POW n=606 babies**	**Standard Care n=619 babies**	**Mean Difference (95% CI)**	**P=**
Apgar score at 5 minutes (mean, SD)	9 (0·04) n=596	9 (0·03) n=613	–0·04 (–0·15, 0·06)	0·41
Number with Apgar <7 at 5 minutes	11 (1·85) n=596	9 (1·47) n=613	0·3 (–1·0, 1·8)	0·61
Arterial cord blood gases taken	307 (51%)	308 (50%) n=614	0·8 (–4·5, 6·4)	0·77
Number with arterial cord Ph <7·05	4 (1%)	7 (2%)	–1·0 (–3·2, 1·2)	0·36
Breastfeeding initiation	300 (51%) n=595	302 (49%) n=615	1·3 (–4·3, 6·9)	0·65
Method of Feeding at discharge	n=584	n=606		
Exclusive breast	285 (49%)	303 (50%)	NA	0·90
Any breast	56 (10%)	55 (9%)		
Bottle	243 (42%)	248 (41%)		
Length of stay in hospital (days)	3 [1, 4]	2 [1.5, 4]	NA	0·75
Admission to NNU	77 (13%)	81 (13%)	–0·04 (–4·2, 3·3)	0·83
Birth weight	n=604	n=616		
Median, IQR	3222 [2870, 3545]	3207 [2805, 3535]	NA	0·27
<1500g	13 (2%)	13 (2%)	NA	0·99
1500–2499g	59 (10%)	59 (10%)		
≥2500g	532 (88%)	544 (88%)		

Gestational age	n=604	n=616		
Median, IQR	40 [38·71, 40·86]	40 [38·71, 40·86]	NA	0·59
<28 weeks	6 (1%)	4 (1%)	NA	0·81
28–31+6 days	8 (15%)	8 (1%)		
32–36+6 days	37 (6%)	32 (5%)		
≥37 weeks	553 (92%)	572 (93%)		
**Neonatal outcomes for those admitted to Neonatal Unit**	**POW n=77**		**Standard Care n=81**	
Birth weight *(grams)*	2585 [1870, 3155], n=77		2800 [1900, 3460], n=80	
Gestational Age *(weeks and days)*	38 [34, 40]		38 [35, 40]	
Reason for admission to NNU				
Congenital abnormality	6 (8%)		8 (10%)	
Feeding problems	1 (1%)		3 (4%)	
Hypoglycaemia	4 (5%)		0 (0%)	
Hypothermia	1 (1%)		2 (2%)	
Infection/sepsis	9 (12%)		22 (27%)	
IUGR	6 (8%)		5 (6%)	
Jaundice	4 (5%)		3 (4%)	
Neurological problems / seizures	0 (0%)		0 (0%)	
Prematurity <28 weeks	9 (12%)		8 (8%)	
Prematurity 28–32 weeks	3 (4%)		4 (5%)	
Prematurity >32 weeks	5 (6%)		10 (12%)	
Respiratory problems	16 (21%)		11 (13%)	
Substance/alcohol misuse	4 (5%)		4 (5%)	
Other	8 (11%)		1 (1%)	
Length of stay in NNU				
Median (IQR)	7 [5, 20]		7 [4, 23]	
Duration of Intensive care (level 3) (days)	0 [0, 3]		0 [0, 2]	
Duration of high dependency care (level 2) (days)	0 [0, 0]		0 [0, 0]	
Duration of special care (level 1) (days)	5 [2,14]		4 [0, 7]	
Duration of transitional care (days)	0 [0, 0]		0 [0, 0]	
Oxygen at 36 weeks post–conceptual age	6 (8%)		5 (6%)	

If yes was this an acute episode?	3 (50%)	4 (80%)
Baby discharged on oxygen?	0 (0%)	0 (0%)
Cerebral US Scan performed during the baby's stay	19 (25%)	15 (19%)
Ventricular dilation >4mm above the 97th centile	1 (1%)	1 (1%)
Intraparenchymal lesion	2 (3%)	1 (1%)
Periventricular leukomalacia	0 (0%)	1 (1%)
Cerebral atrophy	0 (0%)	0 (0%)
Intraventricular haemorrhage (IVH) causing ventricular distension	0 (0%)	2 (2%)
Intraventricular haemorrhage (IVH) Grade I–II	2 (3%)	1 (1%)
Necrotising enterocolitis	0 (0%)	1 (1%)
Stage 1	0 (0%)	0 (0%)
Stage II or III	0 (0%)	1 (1%)
Infection that required either 5 days IV antibiotic only and/or positive neonatal blood culture	27 (35%)	26 (32%)
Retinopathy Of Prematurity (ROP)	0 (0%)	1 (1%)
Stage 1	0 (0%)	0 (0%)
Stage 2	0 (0%)	0 (0%)
Stage 3	0 (0%)	1 (1%)
Stage 4	0 (0%)	0 (0%)
Stage 5	0 (0%)	0 (0%)
Require treatment	0 (0%)	1 (1%)
Death	0 (0%)	1 (1%)
Most likely cause:	NA	IVH prematurity

Values are numbers (percentages) unless otherwise stated.

Supplementary Table 3: Maternal psychological outcomes at 8 weeks after birth

Self-efficacy	POW<br>n=476	Standard Care<br>n=501	Mean Difference<br>(95% CI)	p=
Score	22·39 (0·18)	21·96 (0·17)	0·43 (–0·06, 0·91)	0·08
**Number of social risk factors**				
1 social risk factor	23·0 (0·37) n=123	22·6 (0·32) =153	0·42 (–0·54, 1·38)	0·39
2 or more social risk factors	22·2 (0·21) n=353	21·7 (0·20) =348	0·48 (–0·08, 1·04)	0·09
**Mother-to-infant bonding[1]**	**POW<br>n=457**	**Standard Care<br>n=489**	**Mean Difference<br>(95% CI)**	**p=**
Score	1·42 (0·11)*	1·73 (0·11)	–0·30 (–0·61, 0·00)	0·05
**Number of social risk factors**				
1 social risk factor	1·34 (0·21) n=120	1·55 (0·20) n=152	–0·29 (–0·77, 0·36)	0·48
2 or more social risk factors	1·45 (0·12) n=337	1·81 (0·14) n=337	–0·35 (–0·72, 0·01)	0·06

*One observation missing outcome on mother to infant bonding. Scales range from 0–24 where higher scores indicate worse mother-to-infant bonding
Values are mean, (SE).

Supplementary Table 4: Child Health Assessment and primary immunisation

	POW<br>n=600	Standard care<br>n=613	Difference (99% CI)	P=
Attendance at 6–8 week routine check	n= 464 (77%)	n= 455 (74%)		
Attended 6–8 week check	427 (71%)	405 (66%)	0.03 (–0·02, 0·08)	0·1427
Primary immunisation	n= 581 (97%)	n= 591 (96%)		
Not given	37 (6%)	48 (8%)		0·459
Partial	81 (14%)	75 (12%)		
Complete	463 (77%)	468 (76%)		
Feeding method at 6 weeks	n= 540 (90%)	n= 537 (88%)		
Formula	329 (55%)	346 (56%)		0·501
Mixed	101 (17%)	92 (15%)		
Exclusive breast	110 (18%)	99 (16%)		

Values are numbers (percentages) unless otherwise stated.

Supplementary Table 5: Description of POW contact

POW contact	Total	Antenatally	Postnatally
Total number of contacts	n=17534	n=13516 (77%)	n=4018 (23%)
Median (IQR)	25 [15, 5]	19 [12, 27]	6 [3, 10]
Median face-to-face contacts (IQR)	7 [3, 12]	5 [2, 8]	3 [1, 4]
Method of contact	n=17534	n=13516 (77%)	n=4018 (23%)
Face to face	4667 (27%)	3465 (26%)	1202 (30%)
Phone	4368 (25%)	3449 (26%)	919 (23%)
Other (group, letter, voicemail, spoke to family member, text, referral, discussion with professional, research)	8041 (46%)	6256 (46%)	1785 (44%)
Woman did not attend face to face	451 (3%)	341 (3%)	110 (3%)
Duration of face-to-face contacts	n=4667	n=3465 (74%)	n=1202 (26%)
Under 30 mins	132 (3%)	100 (3%)	32 (3%)
30 mins – 1 hour	1724 (37%)	1310 (38%)	414 (34%)
1-2 hours	2207 (47%)	1595 (46%)	612 (51%)
Over 2 hours	592 (13%)	451 (13%)	141 (12%)
Venue for contact (Face to face or group)	n=4667	n=3465 (74%)	n=1202 (26%)
Woman's home	3925 (84%)	2857 (82%)	1068 (89%)
Other	722 (15%)	593 (17%)	129 (11%)
Duration of other contacts	12860	10046 (78%)	2814 (22%)
Under 30 mins	12048 (94%)	9446 (94%)	2602 (92%)
30 mins or more	802 (6%)	594 (6%)	208 (7%)
Number of women allocated the addition of a POW	n=662	n=662	n=662
Number of women with over 10 face to face contacts	194 (29%)	109 (16%)	9 (1%)
Number of women with contact only by phone or letter	3 (<1%)	3 (<1%)	98 (15%)
Number of women with no contacts	0 (0%)	0 (0%)	0 (0%)
Number of women who disclosed additional social risk*	83 (13%)	77 (12%)	9 (1%)

*Three women disclosed additional social risk both antenatally and postnatally
Values are numbers (percentages) unless otherwise stated.

Supplementary Table 6: Description of type of support given by POW

	**Total**	**Antenatally**	**Postnatally**
Type of support given	n=18888	n=13344 (71%)	n=5544 (29%)
Finance/Benefit/legal	3552 (19%)	2602 (19%)	950 (17%)
Health matters (diet/smoking/lifestyle)	3154 (17%)	2530 (19%)	624 (11%)
Emotional	3262 (17%)	2420 (18%)	842 (15%)
Housing	2865 (15%)	2373 (18%)	492 (9%)
Isolation	1136 (6%)	848 (6%)	288 (5%)
Accompany to appointment	743 (4%)	654 (5%)	89 (2%)
Language problems: reading/writing	694 (4%)	583 (4%)	111 (2%)
Referral to other agency	789 (4%)	541 (4%)	248 (4%)
Social support/child protection	529 (3%)	373 (3%)	156 (3%)
Mental health	389 (2%)	293 (2%)	96 (2%)
Alcohol/Substance misuse/drugs	90 (<1%)	78 (1%)	12 (<1%)
Domestic violence	62 (<1%)	49 (<1%)	13 (<1%)
Breast feeding support	35 (<1%)	N/A	35 (1%)
Breast feeding advice	580 (3%)	N/A	580 (10%)
Bottle feeding support	14 (<1%)	N/A	14 (<1%)
Bottle feeding advice	412 (2%)	N/A	412 (7%)
Baby care support	33 (<1%)	N/A	33 (1%)
Baby care advice	466 (2%)	N/A	466 (8%)
Budgeting	83 (<1%)	N/A	83 (1%)

Values are numbers (percentages) unless otherwise stated.
